# The Value of PD-L1 Expression as Predictive Biomarker in Metastatic Renal Cell Carcinoma Patients: A Meta-Analysis of Randomized Clinical Trials

**DOI:** 10.3390/cancers12071945

**Published:** 2020-07-17

**Authors:** Alberto Carretero-González, David Lora, Isabel Martín Sobrino, Irene Sáez Sanz, María T. Bourlon, Urbano Anido Herranz, Nieves Martínez Chanzá, Daniel Castellano, Guillermo de Velasco

**Affiliations:** 1 Medical Oncology Department, University Hospital 12 de Octubre, 28041 Madrid, Spain; carretero_88@hotmail.com (A.C.-G.); cdanicas@hotmail.com (D.C.); 2 Clinical Research Unit, IMAS12-CIBERESP, University Hospital 12 de Octubre, 28041 Madrid, Spain; david@h12o.es; 3 School of Medicine, Universidad Complutense de Madrid, University Hospital 12 de Octubre, 28040 Madrid, Spain; isabelms95@hotmail.com (I.M.S.); irenesaezsanz@gmail.com (I.S.S.); 4 Hemato-Oncology Department, Instituto Nacional de Ciencias Médicas y Nutrición Salvador Zubirán, 14080 Mexico City, Mexico; maitebourlon@gmail.com; 5 Medical Oncology Department, University Clinical Hospital of Santiago de Compostela, 15076 Santiago de Compostela, Spain; urbanoanido@gmail.com; 6 Medical Oncology Department, Jules Bordet Institute, Université Libre de Bruxelles, 1000 Brussels, Belgium; n.martinezchanza@gmail.com

**Keywords:** renal cell carcinoma, PD-L1, predictive, biomarker, treatment

## Abstract

Immune checkpoint inhibitors (ICIs) are soluble antibodies that have dramatically changed the outcomes including overall survival in a subset of kidney tumors, specifically in renal cell carcinoma (RCC). To date, there is no a single predictive biomarker approved to be used to select the patients that achieve benefit from ICIs targeting. It seems reasonable to analyze whether the programmed death-ligand 1 (PD-L1) expression could be useful. To assess the role of PD-L1 expression as a potential predictive biomarker for benefit of ICIs in RCC patients, we performed a search of randomized clinical trials (RCTs) comparing ICIs (monotherapy or in combination with other therapies) to standard of care in metastatic RCC patients according to PRISMA guidelines. Trials must have included subgroup analyses evaluating the selected outcomes (progression-free survival (PFS) and overall survival (OS)) in different subsets of patients according to PD-L1 expression on tumor samples. Hazard ratios with confidence intervals were used as the measure of efficacy between groups. A total of 4635 patients (six studies) were included (ICIs arm: 2367 patients; standard of care arm: 2268 patients). Globally, PFS and OS results favored ICIs. Differential expression of PD-L1 on tumor samples could select a subset of patients who could benefit more in terms of PFS (those with higher levels; *p*-value for difference between subgroups: <0.0001) but it did not seem to impact in OS results (*p*-value for difference: 0.63). As different methods to assess PD-L1 positivity were used among trials, this heterogeneity could have an influence on the results. PD-L1 could represent a biomarker to test PFS in clinical trials but its value for OS is less clear. In this meta-analysis, the usefulness of PD-L1 expression as a predictive biomarker to select treatment in metastatic RCC patients was not clearly shown.

## 1. Introduction

Kidney cancer represents about 5% and 3% of all solid tumors in adults in men and women, respectively [1]. Renal cell carcinomas (RCC), considering only parenchymal tumors and excluding urothelial tumors, involve 80% of all renal malignancies. Metastatic disease is found in 20% of patients at diagnosis. In addition, 25% of those with localized disease will relapse after radical treatment. The expected 5-year overall survival (OS) rate for advanced disease is estimated to be around 20% [2].

Antiangiogenic drugs and immunotherapy shape the landscape of treatment for metastatic RCC. Recently, immune checkpoint inhibitors (ICIs) have transformed the management of metastatic clear-cell RCC (ccRCC) [3,4,5,6,7,8]. Specially, monoclonal antibodies directed against programmed cell death 1 (PD-1) or programmed death-ligand 1 (PD-L1) combined with either cytotoxic T-lymphocyte associated protein 4 (CTLA-4) or with tyrosine kinase inhibitors (TKIs) have shown significant survival improvement in the first-line setting [4,5,6,7,8].

Despite the numerous efforts to identify predictive markers, none were robust enough to be implemented in clinical practice for RCC patients. The International Metastatic Renal Cell Carcinoma Database Consortium (IMDC) [9] is one of the most validated scores to characterize the prognosis of metastatic RCC patients [10].

The IMDC criteria have been recognized as an aid to select the most appropriate treatment for patients, specifically in the case of nivolumab (anti-PD-1) and ipilimumab (anti-CTLA-4) combination in the first-line setting [4]. Recently, the score has been included in the guidelines as a treatment selection strategy [11,12]. Nevertheless, it remains unknown which patient subgroups will benefit the most within the IMDC categories with the different combinations.

## 2. PD-L1 Expression: Immunohistochemistry Antibody and Cutoff

PD-L1 expression on tumor tissue has been determined to be present in 25–60% of the patients depending upon the employed assay. Largely, PD-L1 expression has been identified as a negative prognostic factor in metastatic RCC [3]. In addition, high PD-L1 levels are associated with unfavorable outcomes of TKI therapy [13].

First data on expression of PD-L1 in RCC come from early trials. The first anti-PD-1 drugs tested were MDX-1106 (ClinicalTrials.gov Identifier: NCT00441337) in a phase 1 trial that included five metastatic RCC patients and nivolumab, in a phase 1 trial that included 34 metastatic RCC patients. PD-L1 expression was studied on the surface of tumor cells. None of the patients with PD-L1–negative tumors had an objective response [14,15]. In this study PD-L1 expression was studied by the murine antihuman PD-L1 monoclonal antibody 5H1. Tumors were considered PD-L1-positive if ≥5% of tumor cells showed membranous staining with 5H1.

Studies with atezolizumab (anti-PD-L1) have used an anti-human PD-L1 monoclonal antibody (Clone SP142) but instead of analyzing tumor cell, immune cell (IC) were studied (all types of ICs, including macrophages, dendritic cells, and lymphocytes, were counted together). The number of patients IC0 (low to no PD-L1 expression) was about 75% if the cutoff is >5% and around 50% if the cutoff is >1% [8,16].

Most recent trials have used alternative antibodies and different locations within the tumor sample in order to improve the accuracy of the technique. Two main strategies assessing PD-L1 expression include those focusing only on tumor cell expression (e.g., Tumor Proportion Score (TPS)), and those incorporating also immune cells (e.g., Combined Positive Score (CPS)). A rabbit anti-human PD-L1 antibody (clone 28-8) has been used in several studies testing the anti-PD-1 drug nivolumab. Distinctive limits for PD-L1 positivity were also investigated containing cutoffs of 1% and more or equal than 5% [17,18].

## 3. PD-L1 Antibodies: Pharmacokinetics and Pharmacodynamics

After the intravenous administration of anti-PD-1/PD-L1 antibodies, the highest concentration is reached between 1 and 4 h. The pharmacokinetics of these soluble antibodies has been described as linear. The increase of concentration during the peak is dose-proportional [14,15].

Early studies showed that the levels of anti-PD-L1 antibodies in the blood are amplified as well in a dose-dependent manner. The half-life of anti-PD-L1 antibody has been calculated over 2 weeks. Importantly, median PD-L1 receptor use on CD3^+^ peripheral-blood mononuclear cells after anti-PD-1 therapy has been shown to exceed 65%.

## 4. Data on PD-L1 Expression and Anti-PD-1/PD-L1 Treatment

The predictive value of PD-L1 expression as a biomarker in metastatic RCC patients treated with anti-PD-1/PD-L1 therapy remains indeterminate [3,4,5,6,7,8]. Selected studies have shown that either tumor cell or tumor-infiltrating immune cell PD-L1 overexpression is associated with deeper response rates with ICIs across different solid tumors, not only metastatic RCC [19]. Indeed, PD-L1 expression has already been used for treatment selection in solid tumors such as lung cancer [20,21]. Nevertheless, tumors that do not express PD-L1 may benefit from ICI. One theory is that the expression of PD-L2 modifies the response; current available tests do not assess this protein [22].

Trials have explored different efficacy parameters according to PD-L1-expression status at a 5% cutoff. In a randomized trial with nivolumab, the group of patients (27%) with tumors PD-L1 expression ≥5% had a median progression-free survival (PFS) of 4.9 months versus 2.9 months in the PD-L1 < 5% subgroup; overall response rate (ORR) was 31% in the PD-L1 ≥ 5% subgroup and 18% in the PD-L1 < 5% subgroup; median OS was not reached in the PD-L1 ≥ 5% subgroup and 18.2 months in the PD-L1 < 5% subgroup. The authors did not find differences with the cutoff ≥1% for PD-L1 expression [17].

In a phase 1 trial testing atezolizumab, there was no correlation between tumor cell score of PD-L1 and outcomes. However, the subgroup of patients with low-to-no PD-L1 expression (IC0) tended to have worse survival [16]. In the phase 3 JAVELIN Renal 101 trial, the combination of avelumab plus axitinib showed an improvement on median PFS in those patients with at least 1% of immune cells staining positive within the tested tumor area (primary endpoint) [6]. On the other side, in the phase 3 KEYNOTE-426 trial, treatment with pembrolizumab plus axitinib resulted in longer OS and PFS compared to sunitinib and regardless of PD-L1 expression measured by CPS (tumor and immune cells); however there was a trend of a greater benefit in patients with higher PD-L1 expression [5].

## 5. PD-L1 Expression and Duration of Response

There is lack of data showing whether the duration of response is associated with PD-L1 expression [16]. PD-L1 expression is associated with high expression of the T-effector (Teff) gene signature and CD8^+^ T cell infiltration. The predictive relevance of PD-L1 expression on IC is further supported by the strong correlation of PD-L1 IC as determined by immunohistochemistry with the Teff immune gene signature.

## 6. PD-L1 Expression: Differences in Primary Tumor Versus Metastases

PD-L1 expression has been associated with poor pathologic features and high nuclear grade areas. Discrepancies in tumor cell PD-L1 expression by immunohistochemistry between primary tumors and metastases counterparts have been described in around 20% of metastatic RCC patients. Based on published data, the PD-L1 expression in the primary tumor seems more common than in the metastases. Largely, PD-L1 expression in multiple metastases from the same primary tumor is consistent [23]. These fluctuations could be predisposed by the particular tumor microenvironment and hypoxia status in each individual tumor location [24,25].

## 7. PD-L1 Expression: Differences Localized Versus Metastatic Tumors

In RCC, increased PD-L1 expression has been found to be considerably associated with large tumor size and TNM stage [26]. In a series of 194 nephrectomies from the Mayo Clinic, 33% of RCC patients were PD-L1 negative [27]. Slightly higher data are presented in a recent pivotal trial ranging from 25% to 40% of PD-L1 negative patients.

## 8. PD-L1 Expression: Evolution with Treatment

In a phase 2 trial with nivolumab, PD-L1 expression on tumor cells was assessed by immunohistochemistry in fresh biopsies obtained at baseline and at the second cycle. There was no consistent change in tumor PD-L1 expression following nivolumab treatment relative to baseline [18]. While baseline PD-L1 expression by immunohistochemistry did not correlate with response to atezolizumab in combination with bevacizumab, upregulation of PD-L1 was only detected in one patient, who demonstrated a partial response on a phase 1 trial [28].

## 9. Gene Expression Profiles

Different studies have shown that tumors with an angiogenic signature present an enhanced response to tyrosine kinase inhibitors such as sunitinib, whereas tumors with a Teff gene signature had better correlation with higher PD-L1 expression, CD8^+^ T cell infiltration, and better response to anti-PD-L1 treatment. Interestingly, a subanalysis of a randomized clinical trial showed that a myeloid inflammatory signature (a subgroup with Teff gene signature) benefited from receiving the combination treatment with an anti-Vascular Endothelial Growth Factor (VEGF) antibody (bevacizumab) and an anti-PD-L1 antibody (atezolizumab) while presenting a poor response to atezolizumab in monotherapy [8].

In the Javelin 101 trial, an association between a signature with 26 gene (including several genes implicated in T cell signaling, proliferation, chemokine expression, and other immune response genes) and improved PFS was observed with the combination of axitinib (TKI) and avelumab (anti-PD-L1). However, this association was not observed with sunitinib monotherapy [6].

## 10. Other Potential Biomarkers

Tumor mutational or tumor neoantigen burden have arisen as promising biomarkers for response to ICIs. The evidence seems to be solid in lung cancer although the biomarker is not flawless [29]. RCC has been shown to express a high frequency of clonal indel mutations, potentially related to neoantigen abundance and CD8^+^ T cell activation [30]; however the association between these features and response to ICIs in RCC has yet to be confirmed [8].

Although further studies are necessary to confirm the findings, loss-of-function mutations in specific genes such as *PBRM1* might predict clinical response to anti-PD-1 antibodies in metastatic RCC according to whole-exome sequencing studies in patients treated with nivolumab [31,32].

CD8^+^ T cell infiltration has been shown to be an adverse prognostic factor for RCC [33]. Contrary, increased amounts of tumor CD8^+^ T cells have been associated with an improved PFS in those patients treated with axitinib plus avelumab but not in patients treated with sunitnib [6]. CD8^+^ infiltration has been shown to be associated with PD-L1 expression. Further data are needed to determine the value of CD8^+^ T cell density and its relationship with PD-L1 as a biomarker for ICI in RCC.

From a different angle, the microbiome (the genetic material within the microbiota) and its variations could be associated with the benefit of ICIs. The microbiome influences the processes of antitumor immunity, and the variations of some bacterial species have been associated with an increased likelihood of response [34,35]. In fact, studies in RCC have shown that antibiotic use could decrease the response to ICI in RCC [36]. Whether the microbiome may alter PD-L1 expression has not been really studied. Further studies focusing on microbiome manipulation in RCC are ongoing [37].

Finally, liquid biopsy is another promising source of information currently under investigation in RCC. Soluble immune checkpoint-related proteins (including PD-1, PD-L1, and CTLA-4 among others) have been shown to be associated with advanced disease, recurrence, and survival in a study with RCC patients, highlighting the potential prognostic value of these biomarkers [38]. In lung cancer, the molecular characterization of PD-L1 expression in circulating tumor cells (CTC) might be supportive to identify a subgroup of patients that will most likely benefit from ICI therapies [39].

## 11. PD-L1 by Immunohistochemistry as a Biomarker in RCC

Currently, the most valuable biomarker due to availability and worldwide access is the determination of PD-L1 by immunohistochemistry. Due to the current uncertain value for metastatic RCC, we performed a meta-analysis of published randomized clinical trials (RCTs) in order to analyze the predictive role of PD-L1 expression and its potential usefulness in treatment decisions in metastatic RCC patients.

## 12. Material and Methods

### 12.1. Literature Search and Inclusion Criteria

The literature search was accomplished by May 1 2019. Two different databases were reviewed: MEDLINE and EMBASE. Only agents targeting PD-1/PD-L1 approved or extensively studied in RCC were included in the search: (a) anti-PD-1 antibodies: nivolumab (Opdivo^®^), pembrolizumab (Keytruda^®^), (b) anti-PD-L1 antibodies: atezolizumab (Tecentric^®^) and avelumab (Bavencio^®^). The specific words used during the search were (“nivolumab” OR “pembrolizumab” OR “atezolizumab” OR “avelumab” OR “PD-1” OR “PD-L1”) AND (“renal cell carcinoma” OR “RCC” OR “kidney cancer”). Additionally, the manufacturers’ package inserts for drugs included in the meta-analysis were also analyzed to spot original or different data not reported in published trials.

All RCTs that compared ICIs based therapy (either in monotherapy or in combination with another ICI or VEGF-targeted therapy) versus the previous standard of care (TKIs or mammalian Target of Rapamycin (mTOR) inhibitors in monotherapy) in any line of treatment in adults (≥18 years-old) with metastatic ccRCC were included. The review was restricted to RCTs in humans and published in English. Non clear-cell RCC studies were excluded. Every publication was reviewed, but only the most complete report of the RCTs was included when duplicate publications were identified. We tried to decrease the heterogeneity among the results gathering only comparisons of ICIs based therapy (defined previously) with TKIs or mTOR inhibitors in monotherapy; other combinations were excluded. We selected the most validated endpoints for efficacy: PFS and OS. Trials that met the following criteria were included in the meta-analysis: randomized phase II or III trials, prospective clinical studies in patients with metastatic ccRCC, and trials with at least one of the previous efficacy endpoints mentioned above available. Two reviewers (A.C-G. and G.d.V.) independently evaluated studies for eligibility.

### 12.2. Data Extraction and Clinical Endpoints

Two investigators (A.C-G. and G.d.V.) extracted the data individually, discordances were resolved by consensus. Data was reported agreeing to Preferred Reporting Items for Systematic reviews and Meta-Analyses (PRISMA) guidelines [40]. Collected variables included the first author’s surname, year of publication, National Clinical Trials (NCT) registry number, study phase, number of previous treatment lines received, selection of population by PD-L1 expression on tumor samples (yes/no), method employed to assess PD-L1 expression, percentage of PD-L1 considered as a positive result, number of enrolled subjects, number of enrolled patients according to PD-L1 expression status, criteria used for assessing efficacy (Response Evaluation Criteria In Solid Tumors (RECIST) or others), blinding (yes/no), treatment arms, number of patients per treatment arm in the total population, and according to PD-L1 expression subgroups, and median age. The efficacy endpoints selected for the analysis (hazard ratios (HR) with confidence intervals (CI) for PFS and OS between treatment arms) were obtained in the total population and according to PD-L1 expression subgroups when available.

### 12.3. Statistical Analysis

Stata version 16 (https://www.stata.com) was the software used for the main statistical analysis. HRs with CIs were the parameters considered to assess the impact on OS and PFS of treatment based on ICIs as compared to standard of care. I2 statistics were used to assess the study heterogeneity among the included trials; these evaluations estimate the significance of heterogeneity compared to chance in relation to the variation observed across the studies [41]. Random and fixed-effects models were used to pool studies depending on the heterogeneity of the studies included. PD-L1 expression on tumor samples (positive or negative depending on the techniques used in each trial) was the clinical feature employed to perform subgroup analyses. Egger′s test, the test for asymmetry of the funnel plot, was performed to reject publication bias [42]. In addition, as other causes of asymmetry could exist, contour-enhanced meta-analysis and trim-and-fill method were used to distinguish publication bias from these other possibilities [43].

## 13. Results

### 13.1. Study Selection

**Figure 1:**
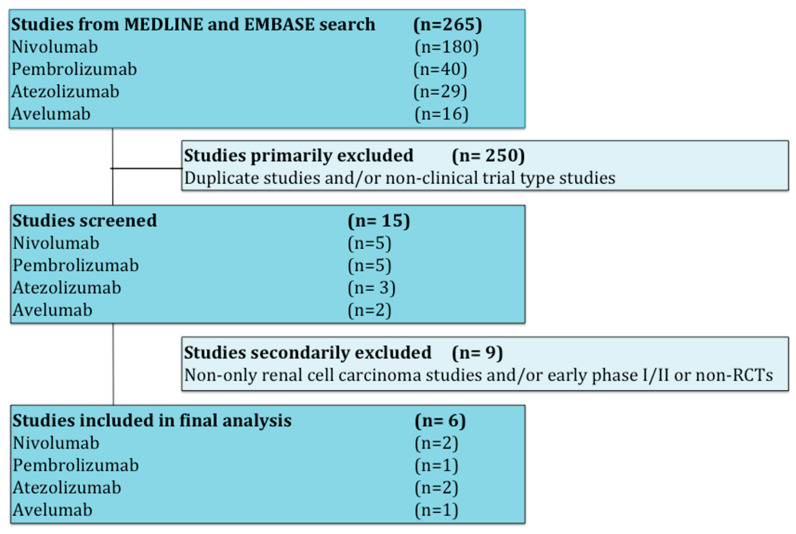
Flow diagram for identification and selection of studies.

Studies that met criteria for the final analysis are shown in the flow chart (Figure 1). A total of 265 studies were reviewed through our screening process for RCTs. We did not include (i) duplicate studies and/or no-clinical trial type studies (*n* = 250) and (ii) no-only renal cell carcinoma studies and/or early phase I/II or non-RCTs (*n* = 9). Six RCTs met the standards for inclusion in the meta-analysis.

The baseline characteristics of each trial are displayed in Table 1. All trials but one [3] were performed in the first-line setting for metastatic RCC (treatment naïve). One trial reported the results only in the intermediate and poor risk-groups according to the IMDC score (https://www.imdconline.com) [4]. All studies had two treatment arms except one, which had three arms [8]. In one study, everolimus (mTOR inhibitor) was the control arm (in the second- and third-line setting) [3]; the remaining studies used sunitinib in monotherapy as the control arm. There were no placebo-controlled trials that met the criteria of the study. None of the studies restricted eligible populations according to PD-L1 expression; different assays and thresholds were used among trials to measure PD-L1 expression by immunochemistry. Only three studies included primary endpoints involving exclusively PD-L1 positive patients [6,7,8] but the rest of them only performed primary endpoints in the intent-to-treat (ITT) population [3,4,5]. A total of 4635 patients were available for the meta-analysis: 2367 patients were assigned to the therapy based on ICIs arm (432 to pembrolizumab plus axitinib, 555 to atezolizumab plus bevacizumab, 103 to atezolizumab in monotherapy, 425 to nivolumab plus ipilimumab, 442 to avelumab plus axitinib, and 410 to nivolumab in monotherapy), and 2268 were assigned to the control arm (1857 received sunitinib and 411 received everolimus). PFS was assessed in all trials according to the RECIST v1.1. All RCTs were sponsored by pharmaceutical companies.

### 13.2. Global PFS and OS Results

Globally, both PFS and OS results favored therapy based on ICIs. The HR for PFS was improved in those patients treated with ICIs compared to standard of care (HR 0.82; 95% CI 0.73–0.92, *p* = 0.0006), as well as the HR for OS (HR 0.73; 95% CI 0.60–0.88, *p* = 0.0012).

Evidence of asymmetry in the studies addressing OS was observed (*p* = 0.0192); by using contour-enhanced meta-analysis funnel plots we can conclude that small studies with negative results have not been published. Evidence of asymmetry in the results of those studies addressing PFS was not verified (*p* = 0.110).

### 13.3. PFS Results According to PD-L1 Expression

**Figure 2:**
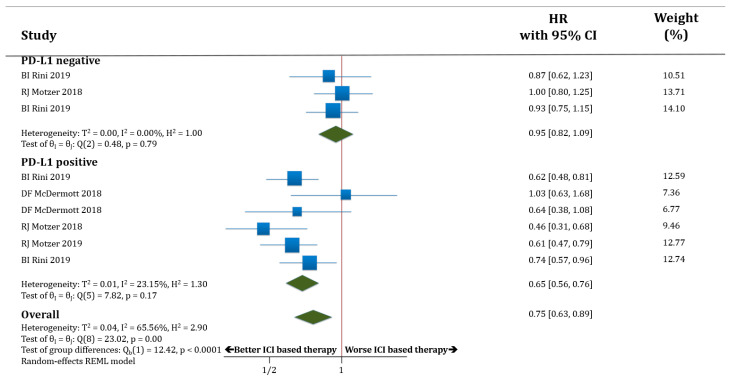
Forest plot diagram: hazard ratio (HR) with 95% confidence interval of progression-free survival (PFS) between arms according to programmed death-ligand 1 (PD-L1) expression subgroups.

In PD-L1 negative patients (only three studies with available information) receiving therapy based on anti-PD-1/PD-L1 antibodies, PFS was not improved compared to those patients receiving standard of care (HR 0.95; 95% CI 0.82–1.09). The PD-L1 positive patients receiving therapy based on anti-PD-1/PD-L1 antibodies had better PFS compared to those patients receiving standard of care (HR 0.65; 95% CI 0.56–0.76). The difference in terms of PFS between these two groups (PD-L1 negative versus PD-L1 positive populations) was statistically significant (*p* < 0.0001; Figure 2).

### 13.4. OS Results According to PD-L1 Expression

**Figure 3:**
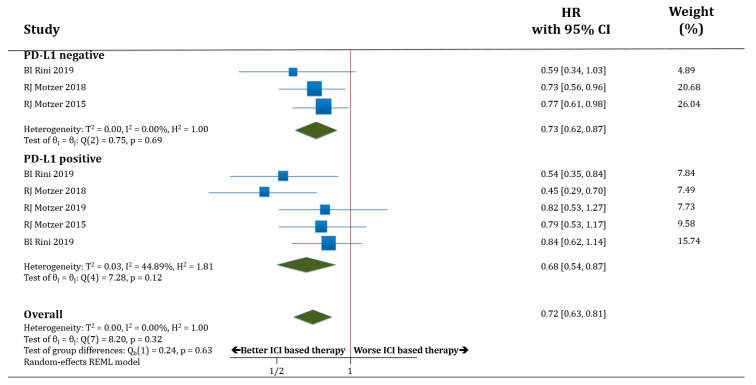
Forest plot diagram: hazard ratio (HR) with 95% confidence interval of overall survival (OS) between arms according to PD-L1 expression subgroups.

In terms of OS, both populations, PD-L1 negative (only three trials with available data) and PD-L1 positive patients, benefited from receiving therapy based on anti-PD-1/PD-L1 antibodies compared to standard of care. HR for OS was improved in PD-L1 negative patients treated with ICIs compared to standard of care (HR 0.73; 95% CI 0.62–0.87) as well as in PD-L1 positive patients (HR 0.68; 95% CI 0.54–0.87). There was no statistically significant difference in OS between the populations PD-L1 negative versus PD-L1 positive patients (*p* = 0.63; Figure 3).

## 14. Discussion

In recent years, the therapeutic landscape of metastatic ccRCC has been broadening as a consequence of the incorporation of different ICIs to the antiangiogenic agents.

Most of the clinical trials were not developed based on predictive biomarkers. Currently, the IMDC score remains as the only useful strategy for treatment selection (restricting the use of the nivolumab plus ipilimumab combination for the intermediate risk- and poor risk-patients) [4]. There are no other validated biomarkers to inform patients about their best treatment choice available in clinical practice. This fact highlights the current lack of personalized therapy in RCC.

The results of this meta-analysis, focused on metastatic RCC, support that PD-L1 expression may be a reliable biomarker for PFS but not for OS. This fact could be relevant for clinical trials design. Therapy based on ICIs improved PFS compared to standard of care in PD-L1 positive patients but not in PD-L1 negative patients. However, OS was improved with immunotherapy regardless of PD-L1 status. Therefore, the role of PD-L1 expression as a predictive biomarker is not flawless and not suitable for selecting the therapeutic strategy. Despite the fact that PFS is considered a validated surrogate endpoint for OS [44] in RCC patients treated with TKIs, its value in those treated with ICIs is less clear, as it has been shown in different trials [3,20]. A long-term “delayed” positive effect has been pointed as a typical characteristic of ICI treatment. This phenomenon, partially reflected by lasting durations of response to ICI therapy, explains the existence of long-term survivor groups with these drugs in different solid tumors even in the absence of benefit in terms of PFS. This fact might help to explain the results obtained in the PD-L1 negative population (benefit in OS but not in PFS). On the other hand, as a consequence of their worse prognosis, PD-L1 positive patients could present shorter median PFS with TKIs compared to PD-L1 negative patients and this feature could explain the difference observed between these groups in terms of PFS but not OS; in this case the positive effect of immunotherapy would manifest earlier in the PD-L1 positive population.

The results obtained in OS should overrule those in PFS, reaching the conclusion that PD-L1 expression has not been shown to be useful for treatment selection in metastatic RCC so far. However, for testing new drugs or combinations targeting PD-1 or PD-L1 it may be useful to consider that PD-L1 expression may predict PFS benefit.

In addition to seeking to optimize and homogenize the assessment of PD-L1 expression among trials, other factors beyond this biomarker that may improve its value are also being studied. Tumor mutational burden, CD8^+^ T lymphocytes infiltration or gene expression profiles could represent alternative biomarkers of response for a better selection of individuals for specific therapies and to optimize the outcomes of this disease [8]. In this sense, gene expression profiles that focus on angiogenic or immunological pathways have shown promising results in patients treated with the combination of atezolizumab plus bevacizumab [8]. In addition, blood constitutes a source for obtaining oncological material and is the basis for the development of liquid biopsy [45]. Due to the improvement of molecular characterization in last years, different nucleic acids as well as exosomes (nanovesicles arisen from endocytosis processes) found in blood may inform about the efficacy of treatments and disease status in a dynamic way [46,47]. As PD-L1 expression is a dynamic biomarker, real time assessment could provide a more accurate evaluation of current status improving the predictive value. As example, in metastatic melanoma patients, it has been shown that expression levels of PD-L1 on circulating exosomes can be modified during treatment with ICIs and those changes might be related to the development of therapy resistance and the type of responses obtained [48].

The future of PD-L1 could be less relevant in the future. This fact will depend on the success of different ICIs being tested or how drugs may change the expression of PD-L1. Bempegaldesleukin (NKTR-214) combined with nivolumab has been shown to change PD-L1 negative tumors (PD-L1 < 1%) to PD-L1 positive tumors (PD-L1 ≥ 1%) [49]. Indeed, several immune checkpoints, such as T cell immunoglobulin 3 (TIM3), lymphocyte activation gene 3 (LAG3), or T cell immunoglobulin and ITIM domain (TIGIT), are potential regulators contributing to the immunosuppressive tumor microenvironment in RCC [50].

A new generation of ICIs, targeting LAG3, VISTA, TIM3, or TIGIT are currently under evaluation. The combination of nivolumab with relatlimab (BMS-986016), an antibody directed against LAG3, has been shown to achieve an ORR of 16% in patients with advanced melanoma progressing after PD-1 or PD-L1 blockade [51]; relatlimab is being tested in a phase III trial (ClinicalTrials.gov Identifier: NCT03470922) [52]. Eftilagimod alpha (IMP321) is a soluble version of LAG3 that preferentially binds to a subset of major histocompatibility complex (MHC) class II molecules and activates antigen-presenting cells. A phase I trial (NCT00351949) tested this drug as monotherapy in 21 metastatic RCC showing both sustained CD8^+^ T cell activation and an increase in the percentage of long-lived effector-memory CD8^+^ T cell in all patients at doses above 6 mg; seven of eight evaluable patients dosed at 6 mg experienced stable disease at 3 months compared with only three of 11 in the lower dose group (*p* = 0.015) [53]. As a consequence of its good safety profile and demonstration of efficacy, the drug is currently being tested in different solid tumors [54]. INCAGN02385 (monoclonal antibody anti-LAG3) and XmAb^®^22841 (bispecific antibody anti-LAG3 and anti-CTLA-4) are also drugs being tested as monotherapy or in combination with anti-PD-1 drugs in a variety of solid tumors including RCC [55,56]. Early-phase trials of anti-TIM3 and anti-TIGIT antibodies are also ongoing in multiple tumor types [57,58,59]. INCAGN02390 (as monotherapy) and MBG453 (as monotherapy or in combination with anti-PD-1) are, both of them, examples of anti-TIM3 antibodies currently being tested in RCC among other tumors [60,61].

OX40 (also known as CD134) is a secondary co-stimulatory molecule whose expression is dependent on full activation of the T cell. OX40 agonists, such as PF-04518600 (in combination with axitinib or avelumab) are other types of molecules undergoing clinical development in RCC [62,63].

Finally, high-dose IL-2 has long been used in RCC as a promoter of T cell proliferation and activation and it is known that a subgroup of patients may achieve complete responses with this therapy [64]. Newer formulations of IL-2 are being developed, such as bempegaldesleukin that is currently being evaluated in combination with nivolumab in RCC in a phase III trial (NCT03729245) [65].

Combination of ICI with cancer cell vaccines, oncolytic viruses, or chimeric antigen receptor CAR T cells is also biologically plausible.

The study has numerous limitations. Primarily, there is not a unique standardized method to assess PD-L1 regardless of the tumor type and potential equivalence among the different techniques is unknown at this moment. Different antibodies, thresholds, and evaluated cells are used to consider the positivity for this marker on the tumor samples with very heterogeneous results. For all these reasons, conclusions obtained from different clinical trials in relation to the role of PD-L1 as a predictive biomarker in ccRCC have to be confirmed.

Patient-level data was not accessible, but trial-level and patient-level meta-analyses may eventually reach comparable conclusions [66]. Finally, response rates (due to lack of data) were not studied. This could be relevant because it is possible that higher PD-L1 expression could be associated with higher probability of response; as this parameter is related to OS, it would be interesting to study it thoroughly and assess its relevance as an endpoint for clinical trials.

## 15. Conclusions

In conclusion, in this meta-analysis, PD-L1 expression did not show to be an accurate and solid biomarker to select treatment for ccRCC patients as both groups (PD-L1 negative and PD-L1 positive) benefited from immunotherapy. The improvement of the assessment of PD-L1 expression status and the introduction of new biomarkers are ongoing; hopefully personalization of systemic therapy in RCC will become a reality in the near future.

## Figures and Tables

**Table 1:** Summary of randomized clinical trials included in the meta-analysis.

Author/Year	Phase	Number of Patients (N)	Line of Treatment	PD-L1 Assay	Experimental Arm	Control Arm	Primary Endpoint
B.I. Rini/2019 ^5^	3	861	1	PD-L1 IHC 22C3 pharmDx assay (combined score: <1; ≥1)	Pembrolizumab + Axitinib (*n* = 432; PD-L1 positive: 243)	Sunitinib (*n* = 429; PD-L1 positive: 254)	OS and PFS in the intent-to-treat population
D.F. McDermott/2018 ^8^	2	305	1	PD-L1 on IC by SP142 IHC assay (<1; ≥1)	Atezolizumab (*n* = 103; PD-L1 positive: 54)	Sunitinib (*n* = 101; PD-L1 positive: 60)	PFS in the intent-to-treat and PD-L1 positive populations
					Atezolizumab + Bevacizumab (*n* = 101; PD-L1 positive: 50)	Sunitinib (*n* = 101; PD-L1 positive: 60)	
R.J. Motzer/2018 ^4^	3	847	1 (intermediate risk- and poor risk-groups)	Dako PD-L1 IHC 28-8 pharmDx test (<1; ≥1)	Nivolumab + Ipilimumab (*n* = 425; PD-L1 positive: 100)	Sunitinib (*n* = 422; PD-L1 positive: 114)	OS, objective response rate and PFS in the intermediate risk- and poor risk-patients
R.J. Motzer/2019 ^6^	3	886	1	Ventana PD-L1 (SP263) assay (<1; ≥1)	Avelumab + Axitinib (*n* = 442; PD-L1 positive: 270)	Sunitinib (*n* = 444; PD-L1 positive: 290)	PFS and OS in the PD-L1 positive population
R.J. Motzer/2015 ^3^	3	821	2 and 3	Dako PD-L1 IHC (<1; ≥1)	Nivolumab (*n* = 410; PD-L1 positive: 94)	Everolimus (*n* = 411; PD-L1 positive: 87)	OS in the intent-to-treat population
B.I. Rini/2019 ^7^	3	915	1	VENTANA PD-L1 SP142 assay (<1; ≥1)	Atezolizumab + Bevacizumab (*n* = 454; PD-L1 positive: 178)	Sunitinib (*n* = 461; PD-L1 positive: 184)	PFS in the PD-L1 positive population and OS in the intent-to-treat population
